# Acute cannabis intoxication among the paediatric population

**DOI:** 10.3389/ftox.2025.1558721

**Published:** 2025-04-14

**Authors:** Ginevra Malta, Giuseppe Davide Albano, Gianluca Lavanco, Anna Brancato, Carla Cannizzaro, Antonina Argo, Simona Contorno, Fulvio Plescia, Stefania Zerbo

**Affiliations:** ^1^ Department of Health Promotion, Mother and Child Care, Internal Medicine and Medical Specialties, Institute of Forensic and Legal Medicine, University of Palermo, Palermo, Italy; ^2^ Department of Health Promotion, Mother and Child Care, Internal Medicine and Medical Specialties, Pharmacology Department, University of Palermo, Palermo, Italy

**Keywords:** cannabis, THC, intoxication, paediatric, children, toxicology, medico-legal issues, forensic

## Abstract

This narrative review synthesizes the toxicological, clinical and medico-legal aspects of paediatric cannabis intoxication. By providing a comprehensive overview, it aims to inform future research, guide policymaking, and enhance clinical and toxicological practice in addressing this growing public health concern. The pharmacokinetics of cannabinoid ingestion in children are significantly influenced by the immaturity of their gastrointestinal tract and metabolic enzyme systems, resulting in altered oral bioavailability. Clinical data indicate that Δ9-tetrahydrocannabinol (THC)-related effects in paediatricpaediatric patients typically emerge within 2 hours of ingestion, with more severe symptoms developing within 4 hours. The endocannabinoid system (ECS) undergoes significant developmental changes, with marked differences in cannabinoid receptor expression and distribution across fetal, neonatal, and adult brains. During neurodevelopment, CB1 receptors exhibit unique expression patterns, including transient localization in brainstem regions critical for neurovegetative functions. These developmental dynamics likely explain children’s heightened sensitivity to THC’s neurological and neurovegetative effects, often resulting in more severe outcomes compared to adults. The reliable detection of cannabinoids involves integrating screening methods with confirmatory analytical techniques. Urine immunoassay testing is widely considered an helpful toolto assess a previous exposure, becoming positive within 3–4 h of ingestion. However, this method is prone to false positives. Plasma THC concentration, when measured close to the event, offers valuable insights into the quantity ingested and the correlation between exposure and clinical outcomes in the impairment window. Hair analysis, while useful for distinguishing between acute and chronic use, is susceptible to various biases. The rising incidence of acute cannabis intoxication in children underscores the urgent need for targeted public health interventions and stricter regulatory frameworks. Preventive measures such as child-resistant packaging, public education campaigns, and cannabis use screening during pregnancy are essential to mitigate risks. Clinicians should consider THC exposure in the differential diagnosis of children presenting with unexplained neurological, immune, or metabolic symptoms.

## 1 Introduction

The endocannabinoid system (ECS) is a highly intricate cell-signaling framework that plays a fundamental role in regulating physiological equilibrium. Cannabinoids, which interact with this system, are broadly divided into three main types (Aizpurua-Olaizola et al., 2016). Endogenous cannabinoids, such as anandamide and 2-arachidonoylglycerol, are produced naturally within the body. Phytocannabinoids, including Δ9-tetrahydrocannabinol (THC) and cannabidiol (CBD), are derived from plants. In addition, synthetic cannabinoids are chemically engineered to replicate or amplify the biological activity of their natural counterparts (Aizpurua-Olaizola et al., 2016).

Δ9-tetrahydrocannabinol (THC), the main psychoactive compound found in cannabis, primarily interacts with the endocannabinoid system by acting as a partial agonist at CB1 receptors. This activation is associated with changes in mood, perception, and cognitive processes (Atakan, 2012). On the other hand, cannabidiol (CBD) is a non-psychoactive cannabinoid that has gained significant interest for its potential therapeutic effects, such as anti-inflammatory and anticonvulsant properties. Despite its promising applications, the exact mechanisms through which CBD exerts its effects remain incompletely understood (Atakan, 2012).

Cannabis ranks among the most commonly consumed substances worldwide, with an estimated user base exceeding 180 million individuals (Tweet et al., 2023).

Recent years have witnessed a notable rise in its use, influenced in part by shifts in regulatory policies, especially in countries such as the United States and Canada. These legislative changes have facilitated the legalization of cannabis for medical purposes and, in certain areas, for recreational use as well (Farrelly et al., 2023).

Among adolescents, cannabis remains the most prevalent illicit substance. In Italy, for instance, an estimated 550,000 adolescents aged 15–19 years, representing 22% of the student population, reported using cannabis at least once in 2023. Alarmingly, 70,000 adolescents (2.8%) indicated nearly daily use (20 or more times per month) (Politicheantidroga.Gov, 2024). Therefore, cannabis use by youth is an important social and public health issue.

The changing legal and cultural landscape surrounding cannabis has led to increased availability of cannabis-derived products, including edibles, which are often formulated with high concentrations of THC. Edibles, frequently manufactured in appealing forms such as gummies and baked goods, pose a heightened risk of unintentional paediatricpaediatric exposure (Connor et al., 2021).

Data from 2017 to 2021 reveal a 1,375% increase in reported paediatric cannabis exposures among children under 6 years of age in the United States. These exposures have led to a corresponding rise in emergency department visits for acute cannabis intoxication in paediatric populations (Myran et al., 2024).

Children are particularly vulnerable to the adverse effects of cannabinoids due to their developing neurological and metabolic systems (Stoner et al., 2022). The immature blood-brain barrier and ongoing brain development render paediatric patients more susceptible to neurotoxic effects compared to adults. Furthermore, the pharmacokinetics of THC in children differ significantly, with prolonged half-life and delayed clearance exacerbating the severity of toxicity (Aychman, Goldman, and Kaplan, 2023). Ingesting cannabis edibles introduces additional risks, as the delayed onset of psychoactive effects may prompt consumption of larger quantities before symptoms manifest, often resulting in severe intoxication (Cao et al., 2016).

Diagnosing acute cannabinoid intoxication in paediatric patients presents challenges due to nonspecific symptoms that overlap with other medical conditions, such as viral encephalitis or metabolic disorders. An accurate diagnosis requires a comprehensive history—including an assessment of potential access to cannabis products—and toxicological screening (Corlade-Andrei et al., 2023). Management is primarily supportive, emphasizing the stabilization of vital functions, though severe cases may necessitate intensive care interventions (Corlade-Andrei et al., 2023).

The increasing incidence of paediatric cannabis intoxication highlights the urgent need for preventive measures. Furthermore, acute cannabis intoxication may sometimes mask underlying neglect or other forms of child abuse, underscoring the critical importance of multidisciplinary approaches involving healthcare providers, social services, and judicial authorities. This narrative review synthesizes the toxicological, clinical and medico-legal aspects of paediatric cannabis intoxication. By providing a comprehensive overview, it aims to inform future research, guide policymaking, and enhance clinical and toxicological practice in addressing this growing public health concern.

## 2 Pharmacological and toxicological effects of cannabis on the paediatric population

Cannabis formulations, such as marijuana (dried leaves), hashish (resin), and hashish oil (concentrated resin extract), contain chemical compounds known as cannabinoids, which are responsible for psychoactive and sedative effects through binding to specific receptors in the brain. The two best-studied and well-known cannabinoids are Δ-9 tetrahydrocannabinol (THC) and cannabidiol (CBD), although more than 100 different cannabinoids have been identified.

The potency of cannabis is mainly determined by the THC concentration, its primary psychoactive component. In recent years, THC content in cannabis products has increased considerably, raising concerns about potential public health implications (Chandra et al., 2019; Freeman et al., 2021). In the past, THC concentrations in cannabis preparations ranged from 2% to 4%, but today, levels of the main psychotropic component can exceed 20% (PaediatricStuyt, 2018; Aguilar et al., 2018). This significant increase in THC potency has exacerbated health risks, including a higher likelihood of developing addiction and the onset of cognitive and psychotic disorders, especially in the most vulnerable population groups, such as young consumers (Schmid et al., 2020; Hoch et al., 2024). In addition, the difficulty in accurately determining the THC dosage in edible and concentrated products contributes to increasing the frequency of accidental intoxication and overdose (Kaczor et al., 2021).

Children under five are one of the most vulnerable groups to the risk of unintentional intoxication from ingesting cannabis products. Specifically, for this age group, the estimated risk of ingesting potentially poisonous substances (cannabis and others) is estimated to be around 46.82%. In this regard, the 2015 Annual Report of the American Association of Poison Control Centers’ National Poison Data System (NPDS) showed the most frequent substances involved in paediatric (≤5 years) exposure: cosmetics/personal care products (13.62%), cleaning substances (11.16%), analgesics (9.12%), foreign bodies (6.45%), topical preparations (5.33%). According to this report, the substance categories most frequently involved in paediatric deaths were analgesics (27.8%), batteries (10.42%), fumes, gases, vapors (10.42%), stimulants and street drugs (8.33%), unknown drugs (8.33%) (Gupta et al., 2003; Gummin et al., 2021). These paediatric intoxications are often classified as “involuntary” or “accidental.” However, the term “exploratory” could describe more accurately the nature of such episodes (Richards et al., 2017), given the innate curiosity and desire to explore typical of childhood (Hoy et al., 1999; McCaig, McCaig, and Burt, 1999).

During this period, intoxications tend to be more severe than those in older children, who are more likely to intentionally use cannabis, usually through inhalation (Cheng et al., 2020). Accidental ingestion is very common when children find and consume cannabis products left unattended by adults (Kaczor et al., 2021). The increasing consumption of cannabis among young adults, particularly in edible forms like cookies and candies, raises the risk of unintentional exposure for younger family members (Claudet et al., 2017a; Claudet et al., 2017b; Wong and Baum, 2019). Edible cannabis products, such as sweets and chocolates, are hazardous because they are indistinguishable from regular treats and are often sold in colourful, attractive packaging that appeals to children (Barrus et al., 2016; Ompad et al., 2022).

Besides unintentional ingestion, passive (or secondhand) marijuana smoke exposure is also a growing concern, especially as children may be more vulnerable due to their higher respiration rates and potential exposure to cannabis products containing elevated THC concentrations (Wang, 2017). Common symptoms of paediatric cannabis exposure include drowsiness, tachycardia, ataxia, and vomiting. More serious effects, like hypotension, coma, respiratory depression, and seizure, occur in less than 3.5% of cases, with some requiring intensive care and invasive measures such as intubation (Leubitz et al., 2021; Tweet et al., 2023).

A recent retrospective review of children under 6 years old presenting with edible cannabis ingestions found that THC dose strongly correlates with toxicity severity. THC doses of 1.7 mg/kg were found to be predictive of severe and prolonged toxicity, providing a threshold for guiding medical interventions and preventive regulations (Pepin et al., 2023).

Paediatric risk stratification is crucial for determining the degree of illness. Indeed, children exhibit different pharmacological effects from THC exposure compared to adults, whether the exposure is intentional or accidental (Stoner et al., 2022). This can be attributed to THC’s unique pharmacokinetic and pharmacodynamic properties (Mehamha et al., 2021). In general, currently available pharmacokinetic and pharmacodynamic data were obtained from studies in healthy volunteers or cannabis users. These data cannot simply be extrapolated to more vulnerable patient groups since paediatric patient-specific variables (low body weight, variable fat percentage, developmental processes) may strongly influence cannabinoid pharmacology.

### 2.1 Pharmacokinetics profile and considerations in paediatric patients

The pharmacokinetics and the effects of cannabinoids depend on the formulation and route of administration, with limited data available for the paediatric population.

THC is a highly lipophilic compound, with almost complete absorption via inhalation, allowing it to quickly enter the bloodstream (within 3–10 min) at concentrations of 10%–35% (Grotenhermen, 2003). Oral THC intake, however, results in slower and irregular absorption, with peak plasma concentrations reached after about 2 hours, at roughly one-tenth the level of inhalation (Lucas, Galettis, and Schneider, 2018). This is largely due to the liver’s first-pass metabolism, which converts THC into an active metabolite, 11-hydroxy-THC (Chayasirisobhon, 2021), which can reach concentrations three times higher through inhalation (Schwilke et al., 2009). The metabolic process leading to the transformation of THC into 11-Hydroxy-THC is produced by the enzymatic system of cytochrome P450 isoenzymes CYP2C9, CYP2C19, and CYP3A4 (Matsunaga et al., 1995; Watanabe et al., 2007; Bansal et al., 2023). Once formed, 11-hydroxy-THC is glucuronidated by the enzyme systems belonging to the family of uridine 5-diphosphoglucuronic acid glucuronyl transferases (UGTs), isoforms 2B7, 2B8 and 2B9, and subsequently excreted through faeces and urine (Schwope et al., 2011). Metabolism also occurs in the small intestine, brain, and extrahepatic tissues expressing the CYP450 enzyme system (Huestis, 2007). Estimates of the elimination half-life of THC indicate a long terminal half-life (22 h), influenced by the equilibration between lipid storage compartments and the blood (Lucas, Galettis, and Schneider, 2018).

Notably, the pharmacokinetic profile of cannabinoid ingestion in the child is influenced by the immaturity of both the gastrointestinal system and the metabolic enzymes, which could impact the oral bioavailability of drugs. With the increased diffusion of high-THC cannabis preparation and the smaller body mass in children, toddler cannabis ingestion results in high serum THC levels despite a small amount ingested (Mehamha et al., 2021). In children, the immaturity of the GI system plays a significant role in altering cannabinoid pharmacokinetics.

Variations in gastric pH, enzyme activity, and intestinal transport mechanisms in neonates and infants could influence oral THC absorption and metabolism. The gastric pH at birth is less acidic (more alkaline) than it is in adults, potentially influencing solubility and degradation of THC in the stomach and, thereby, possibly leading to alternative bioavailability (Kearns et al., 2003; Hsu et al, 2022). In addition, intestinal motility and gastric emptying are immature in neonates, thus absorption is delayed and transit time is longer, potentially leading to variable and longer peak plasma concentrations of THC after oral administration (Allegaert et al., 2018). Enzyme activity in the GI tract is also controlled developmentally. Major drug-metabolizing enzyme expression such as CYP3A4 is significantly lower at birth, reaching only 30%–40% of adult levels in newborns (Lu and Rosenbaum, 2014). This may result in decreased first-pass metabolism of THC in the gut, leading to increased systemic exposure to the parent compound before hepatic metabolism. Further, reduced function of efflux transporters, i.e., P-glycoprotein (P-gp) and breast cancer resistance protein (BCRP), that are involved in drug absorption and excretion at the intestinal barrier, can be another cause of altered THC bioavailability in early life (Alcorn et al., 2019; Ayorinde et al., 2021).

These developmental differences have important clinical implications. The reduced metabolic capacity of the neonatal liver and intestine, combined with increased intestinal permeability and altered enzyme activity, can result in prolonged systemic circulation of THC and its active metabolite, 11-hydroxy-THC. This, in turn, can potentiate the central nervous system effects of cannabis exposure in children, resulting in increased susceptibility to toxicity as well as prolonged clinical presentations (Kearns et al., 2003; Lu and Rosenbaum, 2014).

Overall, clinical reports indicate that paediatric patients develop THC effects within 2 hours of ingestion and severe effects within 4 hours (Lu and Rosenbaum, 2014). Thus, asymptomatic children post-ingestion could likely be monitored for evolving clinical effects.

The distribution of THC in paediatric patients also differs due to higher body fat percentages in early life, with decreases during childhood (Kuzawa, 1998; Butte et al., 2000), and the immaturity of the blood-brain barrier, which may result in higher brain concentration of THC in infants (Goasdoué et al., 2017).

As already presented herein before, besides absorption and distribution, cannabinoid metabolism may also change in paediatrics. THC undergoes extensive hepatic metabolism. Recent studies have shown that the expression of enzyme systems responsible for phase I and II metabolism is closely linked to age, with each system following its own development model (Kearns et al., 2003; Dotta and Chukhlantseva, 2012). In the paediatric population, for example, the expression of CYP3A4, 2C9 and C19 (Lu and Rosenbaum, 2014), crucial for the metabolism of many drugs, is significantly reduced in the first years of life. However, this expression gradually increases with age, reaching levels similar to those found in adults during the first years of development. At the same time, the enzyme UGT2B7, involved in glucuronidation, also shows a slower growth, reaching full expression only after the first years of life (Lu and Rosenbaum, 2014). In this context, reduced expression of UGT2B7 may result in a decreased ability to glucuronide active metabolites of THC, potentially amplifying psychoactive effects and increasing sensitivity to toxic impacts.

In paediatric patients, pharmacokinetic parameters influenced by age, including the variability in the expression of CYP and UGT enzyme isoforms during growth, can result in an increased central nervous system (CNS) distribution of THC and prolongation of half-life (Lu and Rosenbaum, 2014). This has important clinical implications, helping to explain in part the increased susceptibility of children to the toxicological effects of cannabis-containing products (Lu and Rosenbaum, 2014). Thus, those presenting severe effects might also display a prolonged clinical course. A recent investigation highlights that all patients with severe toxicity require several hours to return to baseline (up to 29.53 h) (Pepin et al., 2023). This relationship between age and the pharmacokinetics of THC emphasizes the increased risk for injury that exists in the developing paediatric brain with regard to the important roles of the endocannabinoid system (ECS) in the regulation of key processes of neurodevelopment. The oral bioavailability of CBD is relatively low, ranging between 13% and 19%. It undergoes significant first-pass metabolism, with the majority of its metabolites eliminated through the kidneys. In animal studies, plasma and brain concentrations of CBD correlate with dosage, and its bioavailability can be enhanced using lipid-based formulations. However, despite its widespread use in humans, available data on its pharmacokinetics remain limited (Millar et al., 2018). A recent review summarized the pharmacokinetic factors of CBD ingestion in humans. According to the selected studies in this review, peak plasma concentrations and area under the curve are dose-dependent. Cmax is increased and reached faster in case od oral assumption in a fed state. Moreover, the plasma concentration of CBD was higher if the administration was together with food or in a fed state (Millar et al., 2018). There are limited data about pharmacokinetics and pharmacodynamics of CBD in pediatric patients (Wheless et al., 2019). A clinical trial on the effects of CBD treatment on 34 patients with Dravet Syndrome (5- to 20-mg/kg/day doses) reported adverse events in three cases (pyrexia, somnolence, decreased appetite, sedation, vomiting, ataxia and abnormal behavior (Devinsky et al., 2018; Wheless et al., 2019). A recent study evaluated the effects of CBD therapy in a pediatric population with refractory epileptic seizures (61 patients enrolled). All the oral CBD solutions were generally well tolerated. The most frequent adverse events were somnolence (21.3%), anemia (18%) and diarrhea (16.4%) (Wheless et al., 2019). Another study characterized the pharmacokinetics of a CBD oral production in a small cohort of 12 patients with refractory epileptic encephalopathy. Consistent with earlier findings in children, the majority of patients (83%) exhibited minimal fluctuations in CBD plasma levels, suggesting a zero-order absorption process like that observed with the oral administration of an extended-release drug delivery system (Cáceres Guido et al., 2021).

### 2.2 Pharmacodynamics profile and considerations in paediatric patients

THC toxicity in young children is not only dose-dependent but also influenced by the immaturity of the brain synapses and the ECS. The effects can be variable and may present as a stimulant, hallucinogenic, or sedative response (Lin et al., 2022). Unlike older children, children less than 6 years of age often present with altered sensorium related to encephalopathy (Tweet et al., 2023). This is usually manifested in dilated, sluggish pupils, injected conjunctiva, and euphoria, and states vary from stupor to coma. Autonomic instability, also common, presents as high or low blood pressure, often associated with tachycardia (Claudet et al., 2017a; Claudet et al., 2017b). Moreover, respiratory depression may feature, along with gastrointestinal disturbances such as nausea, vomiting, hyperphagia, dry mouth, and thirst (Richards et al., 2017; Wong and Baum, 2019; Kaczor et al., 2021).

These symptoms are mainly due to the action of THC on the ECS, responsible not only for the known psychoactive properties of the substance, such as euphoria, the feeling of wellbeing, mood tone, analgesia, muscle relaxation, and increased appetite but also for the occurrence of the neurotoxic effects, including drowsiness, motor incoordination, attention deficits, to more severe conditions such as psychosis, anxiety disorders, hallucinations and tachycardia (Crippa et al., 2018).

Cannabinoid effects are mainly attributable to the activation of CB1 receptors (CB1Rs), the abundance of which in the central nervous system (CNS) allows them to modulate different physiological functions (Zou and Kumar 2018). Notably, most, but not all, toxicologically relevant cannabimimetic responses, including the marked reduction in an individual’s ability to control physical and mental functions, are mediated by CB1Rs (Stella et al., 2023).

As a matter of fact, THC exerts the characteristic “tetrad” effects, which refers to four easy-to-measure physiological and behavioral hypo-locomotion, hypothermia, catalepsy, and antinociception, observed with other CB1R agonists. Yet, THC displays lower maximal effect sizes than full receptor agonists, indicating that THC is a partial agonist (Pertwee and Cascio, 2014). On the other hand, CBD does not induce the tetrad effects and has negative allosteric modulatory activity at CB1R, thus counterbalancing THC intoxicating and psychotomimetic effects (Stella et al., 2023).

Previous evidence indicates that the endogenous cannabinoid system plays a critical role in brain development from early gestation. In rodent models, the ECS is already present in the brain’s early stages, influencing key developmental processes (Bara et al., 2021). However, our knowledge of human development remains limited. Although a few studies have begun to investigate the appearance and distribution of cannabinoid receptors during fetal brain development, this area remains understudied (Mailleux and Vanderhaeghen, 1992; Glass, Dragunow, and Faull, 1997; Biegon and Kerman, 2001; Buckley et al., 1998; Zurolo et al., 2010).

Notably, CB1Rs in the human brain are already present and functional as early as the ninth week of gestation, a period concurrent with the beginning of cortical development (Bara et al., 2021). In rodents, a parallel expression is observed by day 11 of gestation (Campolongo et al., 2011). The transient presence on the neuronal fibres in white matter during these stages suggests important roles in axonal growth and neuronal migration. This process likely facilitates the establishment of neuronal pathways, either through direct actions on axons or by influencing the activity of non-neuronal cells like astrocytes and oligodendrocytes, which are critical for guiding these developmental processes (Biegon and Kerman, 2001; Buckley et al., 1998; Zurolo et al., 2010).

In the same way, another study has explored CB1R’s expression throughout the different areas of the developing human brain by ^[3H]^CP55 940 autoradiography, revealed a significant density of CB1Rs at 19 weeks gestation in human fetal brains, particularly in regions where these receptors are also found in adults (Mato, Del Olmo, and Pazos, 2003). This study also demonstrated that these receptors are functionally coupled to signal transduction mechanisms from early prenatal stages, highlighting their active involvement in early neurodevelopmental processes. This early expression suggests that the endocannabinoid system plays a fundamental role in critical events shaping neural development, including synaptogenesis and neurogenesis.

From a paediatric perspective, understanding the early involvement of the ECS is vital, and its dysregulation could potentially contribute to developmental disorders or influence the effects of exogenous cannabinoids when used in therapeutic settings. The ECS is involved in various developmental stages, including neuronal migration, synaptic pruning, and the formation of synaptic connections (Berghuis et al., 2007; Maccarrone et al., 2014; Mulder et al., 2008). The CB1Rs, along with CB2Rs, is part of the seven transmembrane G protein-coupled receptors (GPCRs) family and interacts with endogenous ligands such as anandamide (AEA) and 2-arachidonoylglycerol (2-AG), whose levels are dynamically regulated by synthesis and degradation enzymes (Piomelli, 2003; Kano et al., 2009). Importantly, CB1Rs are highly expressed in the brain from prenatal stages to adolescence, highlighting their role in the maturation of crucial brain structures, including the hippocampus, basal ganglia, and prefrontal cortex (Bara et al., 2021; Mackie, 2025). These regions are essential for higher cognitive functions, emotional regulation, and motor control and are particularly vulnerable during paediatric development. Detailed studies have shown that CB1 receptors are widely distributed across neurons, glial cells, and microglia (Kano et al., 2009; Han et al., 2012; Ilyasov et al., 2018; Araujo, Tjoa, and Saijo, 2019), indicating that the ECS not only modulates neurotransmission but also may influence immune responses in the developing brain.

The CB1R is also present in the neurons of the major neurotransmitter systems, including glutamatergic, gamma-Aminobutyric acid (GABA)ergic, serotoninergic, noradrenergic, and cholinergic neurons (Lutz et al., 2015). Moreover, recent research suggests that CB1Rs may also be expressed in dopaminergic neurons (Han et al., 2023; Baddenhausen, Lutz, and Hofmann, 2024; Oliveira da Cruz et al., 2020), potentially influencing dopamine regulation during critical periods of brain maturation. As CB1Rs regulate the release of key neurotransmitters such as glutamate, GABA, and dopamine, they play a central role in synaptic plasticity, which is essential for learning and memory (Howlett et al., 2002; Piomelli, 2003; Lovinger, 2008; Jutras-Aswad et al., 2009; Katona and Freund, 2012).

Exposure to THC can induce cognitive impairment by acting particularly on CB1Rs expressed in the medial prefrontal cortex (PFC) and hippocampus (Castelli et al., 2023a; Castelli et al., 2023b; Castelli et al., 2024; Brancato et al., 2020; Fernández-Moncada et al., 2024). Activation of CB1R by THC disrupts the balance between excitatory and inhibitory neurotransmission, particularly affecting GABAergic and glutamatergic systems (Castelli et al., 2023a; Castelli et al., 2023b; Castelli et al., 2024; Brancato et al., 2020). This leads to altered synaptic plasticity, impairing memory processes and cognitive functions (Castelli et al., 2023a; Castelli et al., 2023b; Castelli et al., 2024; Brancato et al., 2020). Specifically, THC has been demonstrated to reduce neuronal spiking patterns and synchrony, especially within the hippocampus (Castelli et al., 2023a; Castelli et al., 2023b; Castelli et al., 2024; Brancato et al., 2020. This brain structure is crucially involved in memory encoding and retrieval processes. These mechanisms underline the wide range of cognitive deficits reported for THC, from impaired working memory to attention (Stella et al., 2023).

**FIGURE 1:**
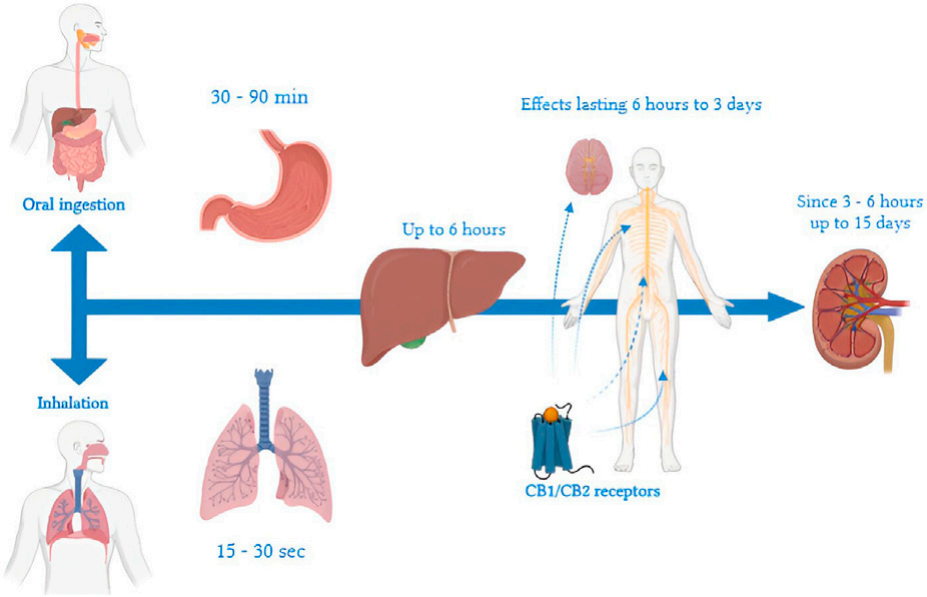
Timeline of THC metabolism: a representation of key steps from ingestion or inhalation, through distribution and hepatic metabolism, to final elimination. Timelines vary by route of administration, frequency of use, and individual characteristics. When inhaled, effects appear within 15–30 s due to rapid pulmonary absorption and direct entry into the systemic circulation. In contrast, ingestion leads to a delayed onset of gastrointestinal effects, which manifest after 30–90 min because of first-pass metabolism in the liver. In the liver, THC is metabolized primarily into 11-OH-THC, an active metabolite, and other inactive compounds, with hepatic action lasting up to 6 h. In the central nervous system, THC binds to CB1 receptors of the endocannabinoid system, producing effects that may persist from 6 h to 3 days, depending on the dosage and frequency of use. THC metabolites, such as THC-COOH, typically become detectable in urine within 1–3 h after use and can remain present for up to 30 days in chronic users. Renal elimination of THC metabolites can continue for up to 15 days, involving residual interactions with CB2 receptors associated with immune functions.

Besides a large distribution in the mammalian brain, CB1Rs are also expressed in the spinal cord, peripheral nerve terminals, cardiovascular and respiratory system, gastrointestinal tract, skeletal muscle, adipose tissue, pancreas and liver (Dallabrida et al., 2024) (Figure 1).

In contrast, CB2Rs are predominantly distributed across peripheral systems like immune cells, the gastrointestinal tract, and the cardiovascular system, with their presence in the central nervous system largely localised to microglia; they regulate neuroinflammatory processes (Cabral et al., 2008).

In addition, THC-cannabinoid receptor binding inhibits neurotransmitters in the autonomic nervous system, such as acetylcholine, noradrenaline, dopamine, serotonin, and GABA (Brunt and Bossong, 2022). Observed clinical effects are both dose- and time-dependent and, therefore, are related to the route of delivery, whether inhaled or oral. In this regard, research has delineated a complex interaction between the ECS and the autonomic nervous system, cardiac conduction, and circulation (Richards, 2020).

CBRs are thought to impact the cardiovascular system through both central and peripheral mechanisms. For example, cannabinoid receptors are also found in the nucleus tractus solitarii of the medulla, where cardiac baroreceptor and chemoreceptor afferent nerves terminate. CNS administration of cannabinoids into the medulla evokes sympathoexcitatory and vagal inhibitory responses, which can be prevented by CB1 antagonism (Gardiner et al., 2001; Niederhoffer and Szabo, 1999).

In humans, the primary cardiovascular effects of exogenous cannabinoids are related to CB1 receptor-dependent increase in heart rate, accompanied by a small increase in blood pressure and cardiac output, which is attributed to an increase in cardiac sympathetic nerve activity This is indicated by a substantial rise in serum norepinephrine concentration at 30 min after cannabis exposure and the sensitivity to the block of this response by the pretreatment with propranolol and rimonabant, CB1 receptor inverse agonist. The decrease in the parasympathetic nervous system was also suggested since atropine pretreatment resulted in an exaggerated heart rate following cannabis smoking (Subramaniam et al., 2019; Pacher et al., 2006).

However, while low to moderate doses of exogenous cannabinoids cause sympathetic stimulation, which favours cardiac automaticity and may increase the risk for tachyarrhythmias, higher doses drive parasympathetic stimulation, which may predispose to bradyarrhythmias, especially in younger individuals with higher baseline vagal tone (Richards, 2020).

Similarly, cannabinoid receptors modulate respiratory function through both central and peripheral mechanisms (Wiese et al., 2023). In particular, CB1 receptors are highly expressed in the pre-Bötzinger complex, which produces the periodic drive for inspiration (Glass, Dragunow, and Faull, 1997). In the periphery, cannabinoid receptors expressed in airway epithelial cells, bronchi, lung tissue, respiratory endothelium, and axon terminals of airway nerves have a demonstrated role in the control of airway responsiveness (Calignano et al., 2000).

In rodents, central and peripheral administration of cannabinoids in rodents leads to respiratory depression across routes of administration (Laudermilk, Marusich, and Wiley, 2023).

In humans, whereas short-term exposure to cannabis smoke is most frequently associated with bronchodilation, strong activation of cannabinoid signalling by intoxicating cannabinoids may lead to respiratory depression (Tetrault et al., 2007; Zwillich et al., 1978; Alon and Saint-Fleur, 2017; Jinwala and Gupta, 2012; Wong and Baum, 2019).

Thus, the ECS appears to be widely distributed in the body and takes a central position in the fine-tuning of physiological processes, thereby integrated into broad functional networks, both in neural and non-neural tissues, that keep the body in homeostatic set-points (Lutz, 2020; Mechoulam and Parker, 2013; Ligresti, De Petrocellis, and Di Marzo, 2016; Cristino, Bisogno, and Di Marzo, 2020).

Notably, ECS undergoes substantial changes throughout life. In particular, cannabinoid receptors are discretely expressed and distributed in fetal, neonatal, and adult human brains (Dallabrida et al., 2024). The midbrain region in neonates has substantially higher levels of cannabinoid receptors compared to adults, with significant increases observed in areas such as the substantia nigra pars reticulata, red nucleus, central grey, and superior colliculus (Glass, Dragunow, and Faull, 1997). Notably, corticolimbic CB1 receptor expression peaks in early life, mainly in the striatum and prefrontal cortex, and stabilises in adulthood (Long et al., 2012). In contrast, CB1 receptor expression reduction in sensorimotor cortices occurs only after adolescence (Heng et al., 2011). The decrease in CB1R expression coincides with cognitive maturation, indicating a possible attenuation of control over inhibitory and excitatory neurotransmissions mediated by this receptor.

Interestingly, during neurodevelopment, CB1 receptors are atypically located in the brainstem (Berrendero et al., 1998), indicating that an age-dependent transient CB1 receptor expression occurs in brain regions relevant for neurovegetative control. Of note, the CB2R expression profile throughout life remains poorly explored (Dallabrida et al., 2024).

Overall, the dynamic changes in cannabinoid receptor distribution during brain development might explain the greater vulnerability of children to the neurological and neurovegetative effects of THC, with potentially more severe symptoms than adults.

Pharmacological interventions targeting ECS activity aim to normalise such pathophysiological processes, thereby rescuing the subject from unfavorable allostatic set-points (Cinar, Iyer, and Kunos, 2020; Chicca, Arena, and Manera, 2016; Tomaselli and Vallée, 2019). This topic will be discussed in more detail in Sections 3.4, 3.5.

This is a challenge that clinicians and public health professionals find increasingly daunting, requiring education, prevention, and policy interventions. Toxicological effects of THC exposure in paediatric populations include both central and peripheral systems, the outcomes of which have been extending into adolescence and adulthood. Greater availability and societal perceptions of safety about cannabis products increase the risks for paediatric exposure. Consequently, vigilance during clinical practice is paramount for the diagnosis and treatment of toxicities related to THC in children.

## 3 Clinical features

**FIGURE 2:**
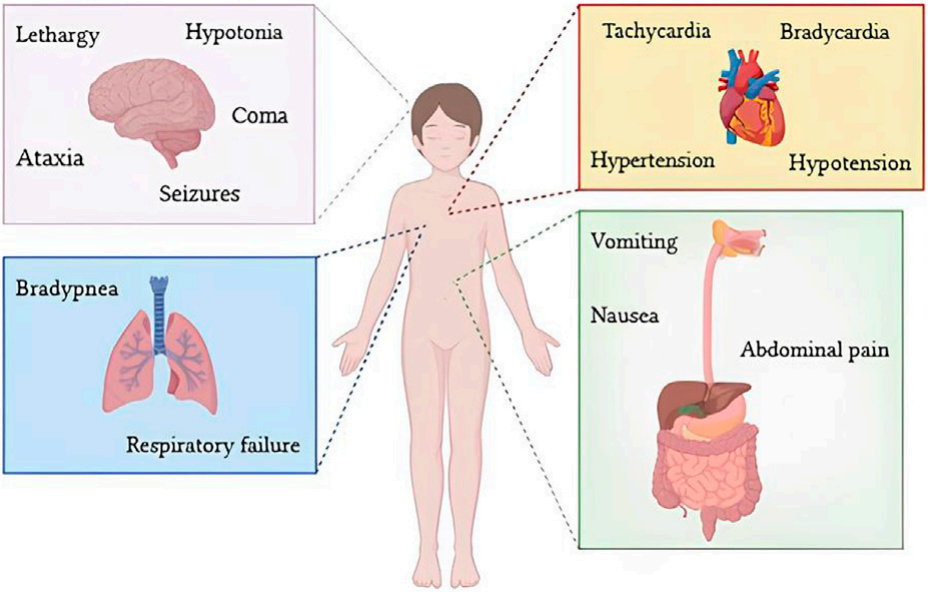
Clinical manifestations in case of acute cannabinoid intoxication in paediatric patients.

Acute cannabinoid intoxication in paediatric patients presents with a wide variety of symptoms, predominantly neurological and cardiovascular (Figure 2) (Dubinin et al., 2024). The severity depends on factors such as the type of product ingested, the concentration of THC, and the child’s body weight. Numerous studies have described a wide range of clinical manifestations, ranging from mild neurological symptoms to potentially lethal conditions such as coma and respiratory failure (Richards et al., 2017; Claudet et al., 2017a). Recent studies have analyzed a significant number of cases, highlighting that the most common symptoms include lethargy, present in 71%–83% of exposed children, with a higher prevalence in children under 2 years old, followed by hypotonia and ataxia. Claudet et al. (Claudet et al., 2017b) emphasize that hypotonia often accompanies lethargy, affecting up to 65% of patients. Coma in a severe but less common manifestation, observed in 10%–18% of significant exposures, especially following the ingestion of edibles with high THC concentrations (Harvey et al., 2022).

According to a retrospective study by Vo et al. (2018), patients in a coma often exhibit associated metabolic alterations, such as hypoglycemia or lactic acidosis. Rare but severe, seizures are reported in fewer than 5% of paediatric cases, according to Feliu et al. (2017). These episodes may be associated with high acute doses of THC and require immediate intervention.

Tachycardia is one of the most common cardiovascular signs, present in 73%–83% of paediatric patients exposed to THC, according to Wang et al. (2016). It is often accompanied by hypertension or hypotension, which can worsen clinical conditions in children. In severe cases, bradycardia in also observed.

According to a study by Tsutaoka et al.vomiting and nausea present in 20%–30% of children hospitalized after the ingestion of edibles, often linked to the high THC concentration in these products. THC-rich edibles with high lipid content can prolong THC absorption, intensifying both gastrointestinal and neurological symptoms. (Tsutaoka et al., 2018). Claudet et al. (2017b) report that abdominal pain is less common but occurs in about 10%–15% of cases, generally associated with hyperemesis (cannabinoid hyperemetic syndrome- CHS). CHS is a clinical syndrome characterized by recurrent episodes of intense nausea and vomiting and abdominal pain, which usually develops after years of THC-containing products exposure. However, it might occur in the paediatric population (Alghamdi et al., 2021). The onset of symptoms is also related to the source of assumption: in the case of oral intake the absorbtion is slower than smoking, with a plasma peak after 1–6 h from ingestion and 1–3 h for the appearance of symptoms (Arbouche et al., 2023).

Early identification of symptoms, combined with timely diagnosis, is essential to prevent long-term complications and reduce mortality (Table 1).

**TABLE 1:** Summary of clinical features of children admitted to Paediatric Emergency Department for cannabis intoxication reported by information source.

Authors, year	Age	Sex	Objective findings	Vital signs	Tox screen	Time to resolution	Exposure	Treatment
Richards et al. (2017)	<6 years	Not specified	Lethargy (71%–83%), ataxia, hypotonia	Tachycardia (27%), hypertension (18%)	Positive	24–48 h	Oral (edible)	Supportive care, observation
Claudet et al. (2017a)	<5 years	Not specified	Lethargy, ataxia (65%), coma (18%)	Tachycardia, hypertension, bradypnea	Positive	24–72 h	Oral (edible)	Supportive care, benzodiazepines for seizures
Wang et al. (2016)	<12 years	Male and female (1:1 ratio)	Vomiting (20%–30%)	Tachycardia (80%), bradycardia (10%)	Positive	24–48 h	Oral (not specified)	Hemodynamic monitoring, ventilation if necessary
Vo et al. (2018)	1–4 years	Male predominant	Coma (10%–18%), hypotonia, seizures	Tachycardia, hypotension, bradycardia	Positive	48–72 h	Oral (resin)	ICU, mechanic ventilation
Tsutaoka et al. (2018)	2–6 years	Not specified	Vomiting, lethargy, bradycardia	Tachycardia (20%–30%), hypertension	Not specified	Not specified	Oral (edible)	Activated charcoal, symptomatic care
Gaudet et al. (2024)	<4 years	Not specified	Lethargy, hypotonia, ataxia, vomiting	Tachycardia (27%), bradycardia (<5%), mild hypertension	Positive	24–48 h	Oral (edible), some inhalation	Supportive care; benzodiazepines for seizures; IV fluids for dehydration
Takakuwa and Schears (2021)	0–15 years	Male (62%), female (37%)	Sedation, hypotension, hypoventilation, coma	Tachycardia (55%), hypotension (9%), respiratory depression (<5%)	Positive	6–48 h	Oral (edible), some inhalation	Supportive care; oxygen supplementation for hypoxia; IV fluids for hypotension
Pepin et al. (2023)	<6 years	Not specified	Sedation (85%), hypotonia, seizures (rare <3%)	Tachycardia (43%), mild hypertension (66%)	Positive	20 h (severe toxicity) - 6 h (mild cases)	Oral (edible)	Observation, IV fluids, intubation in extreme cases

### 3.1 Time to resolution

Resolution of symptoms is determined when the patient is noted to return to their baseline state or to exhibit behaviors typical for their age. Severe toxicity cases are further defined as those involving significant cardiovascular complications (such as bradycardia, hypotension, sinus tachycardia requiring vasopressor support or intravenous fluids, or other dysrhythmias), respiratory issues (including respiratory failure, apnea, or the need for supplemental oxygen), or neurological impairments (such as seizures, myoclonus, unresponsiveness, responsiveness only to painful stimuli, or requiring intubation or sedative medication). Age-appropriate vital sign ranges are based on the Paediatric Advanced Life Support (PALS) guidelines (Skellett et al., 2021). An oxygen saturation level below 90%, as measured by pulse oximetry, is categorized as hypoxia, in line with local standards. Prolonged toxicity is defined as taking more than 6 h for the patient to return to their baseline following ingestion. This 6-h threshold was selected as it aligns with the standard duration for observation in an emergency department setting (Pepin et al., 2023; Skellett et al., 2021).

### 3.2 Long-term effects

Recent research has shown that the neuroadaptations resulting from drug use, including cannabis, can have severe and permanent effects on the developing adolescent brain (Bourque and Potvin, 2021; De Salas-Quiroga et al., 2020). Cannabis may cause lasting damage by interfering with the normal development of neural connections, increasing the risk of psychopathologies such as depression and schizophrenia, while also impairing intellectual abilities (Santovecchi et al., 2015). This includes cognitive difficulties, slowed learning capacity, and memory retention issues (Bellamoli et al., 2012). Neuroimaging studies have shown that, compared to non-users, adolescents who use marijuana exhibit the following: reduced thickness of the insula, reduced brain sulci in both hemispheres, and reduced cortical thickness in the right frontal lobe (Lopez-Larson et al., 2011). However, studies conducted to date have not been able to determine whether these brain and cognitive anomalies predate substance use. Cannabis use is associated with an increased risk of early-onset psychosis and an intensification of schizophrenia symptoms. A genetic correlation between cannabis use and the development of psychosis is therefore suspected. However, the relationship between cannabis use and major depressive disorders remains unclear. What emerges is that cannabis use is more prevalent among individuals with major depressive disorder than in the general population (Urits et al., 2020). Future longitudinal studies on adolescents before cannabis exposure could help clarify its influence on brain development (Santovecchi et al., 2015). Long-term cannabis use in paediatric and adolescent populations is associated with significant respiratory, cardiovascular, and systemic effects. Chronic inhalation of cannabis smoke can lead to respiratory issues similar to those seen with tobacco, including chronic bronchitis, airway inflammation, and reduced lung function. Studies indicate an increased risk of wheezing, cough, and mucus production, with potential for long-term lung damage (Muheriwa-Matemba et al., 2024; Hall and Degenhardt, 2014). Cardiovascular effects include elevated heart rate, altered blood pressure regulation, and increased risk of arrhythmias. Cannabis-induced vasodilation can lead to orthostatic hypotension, while prolonged use may contribute to endothelial dysfunction and increased cardiovascular risk later in life (Muheriwa-Matemba et al., 2024). Beyond respiratory and cardiovascular effects, cannabis use in adolescents has been linked to metabolic disturbances, including increased appetite and altered glucose metabolism, which may predispose users to obesity and insulin resistance. Additionally, immune system modulation has been observed, potentially increasing susceptibility to infections (Padoan et al., 2023).

### 3.3 Diagnosis

Diagnosis is based on detailed anamnesis, collecting information about the home environment and verifying the possibility of exposure to cannabis, including edibles, inhalation, or accidental ingestion. Clinical evaluation might identify neurological, respiratory and cardiovascular symptoms such as lethargy or somnolence, altered mental status, ataxia and tachycardia. The toxicological screening (urine drug screen) is a primary diagnostic tool in paediatric cases of cannabis and other substance intoxications (Takakuwa and Schears, 2021). The sensitivity of urine drug screen (UDS) allows for the detection of THC metabolites, amphetamines, opioids, and other drugs commonly involved in accidental exposures. However, Richards et al. (2017) highlighted a significant limitation: UDS identifies the presence of the substance but does not correlate with the dose or clinical severity of the intoxication. For this reason, the test must always be interpreted in combination with anamnesis and clinical signs. Tsutaoka et al. (2018) reported that, in children intoxicated by THC-containing edibles, UDS was positive in almost all tested patients. The authors stressed that toxicological testing is particularly useful for confirming exposure in cases where the clinical history is unclear or caregivers are uncooperative (Gonedes and Eric, 2024).

In more complex paediatric cases, such as patients with altered mental status or seizures, Vo et al. (2018) suggest using computed tomography (CT) scans to rule out other causes, such as cranial trauma or neurological conditions. Additionally, in cases of unexplained neurological symptoms, cerebrospinal fluid (CSF) analysis can help exclude infections such as encephalitis or meningitis, although it is not indicated in the absence of fever, deteriorating mental state or persistent or progressive encephalopathy (Wong and Baum, 2019).

Pélissier et al. (2014) reiterated the importance of detailed anamnesis and physical examination as the first steps in diagnosis. In their analyzed cases, approximately 65% of children with cannabis intoxication presented with lethargy and hypotonia, and the results of toxicological tests were useful in confirming exposure. However, they caution that early diagnosis requires a high index of clinical suspicion, especially in younger children who may be more vulnerable to severe manifestations.

### 3.4 Therapy and management

Secondary to the public magnitude of cannabis use, the emergency physician must provide an early recognition, management, evaluation, and counseling of suspected paediatric unintentional cannabis ingestion. Standard treatment of acute cannabis intoxication is primarily supportive, with a focus on airway, breathing, and circulation, followed by treatment of cannabis-related symptomatology (Wong and Baum, 2019; Richards et al., 2017).

The cornerstone of treatment includes monitoring vital signs, ensuring airway patency, and administering intravenous fluids for dehydration or hypotension (Takakuwa and Schears, 2021). Management depends on the severity of symptoms. Neurological symptoms such as seizures can be initially treated with benzodiazepines and intubation should be considered for unresponsiveness or respiratory failure. Cardiovascular symptoms e.g., hypotension can be treated with intravenous fluids or vasopressors to correct hypovolemia. Oxygen supplementation or mechanical ventilation can be considered in the case of hypoxia or respiratory depression (Kaczor et al., 2021; Pélissier et al., 2014; Richards et al., 2017). Activated charcoal may be administered within a few hours of ingestion, although it is usually not effective (Wang, 2017; Wong and Baum, 2019). In severe cases of cannabis toxicity, Flumazenil, a selective benzodiazepine antagonist, may have some therapeutic effect (Pianca et al., 2017; Richards et al., 2017). Severe complications, such as low GCS scores or coma, might require admission to the psychiatric or intensive care unit (Monte et al., 2017). In cases with cardiac or respiratory complications, these should be managed according to the underlying etiology (Pianca et al., 2017). Consultation with a regional poison control center is encouraged for all symptomatic paediatric cannabis intoxications.

Cannabinoids may be used in the treatment of hyperactivity disorders, anxiety, sleep disorders and self-injurious behaviors in children. The most described effects are: vomiting, fever, drowsiness in case of overdose, sleepiness, fatigue, diarrhea. However the long term effects of CBD are unknown and the research in this field is limited (Kachru et al., 2021).

### 3.5 Emergency management and prognosis

The toxicity is best managed by the emergency department physicians (lead the initial assessment, stabilization and management), toxicologists (guide specific interventions and confirm diagnosis), paediatric neurologists (address complications like seizures or altered mental states) and critical care teams (manage severe cases requiring intensive care unit admission for intubation, sedation, or advanced monitoring) (Burns et al., 2018).

Patients should be observed for 6 h for resolution of symptoms and be admitted for monitoring when there is central nervous system (CNS) depression, altered mental status, multiple seizures, persistently abnormal vital signs or continued hemodynamic instability (Takakuwa and Schears, 2021). Longer periods of toxicity can be expected to occur with ingested edible products and with synthetic cannabinoids. Patients returning to baseline do not require any further testing or follow-up and should be counseled (Monte et al., 2017; Heizer et al., 2018).

The prognosis for cannabinoid intoxication in children is generally favorable with timely intervention. Symptoms typically resolve within 24–48 h, but it can take hours to days for the child to return to baseline depending on the amount ingested (Tweet et al., 2023).

The most common sequelae are transient and reversibile, with no long-term complications reported in mild-to-moderate cases. However, recurrent intoxications may indicate neglect or household exposure to cannabis, warranting a social work and legal evaluation (Richards et al., 2017; Stoner et al., 2022).

**FIGURE 3:**
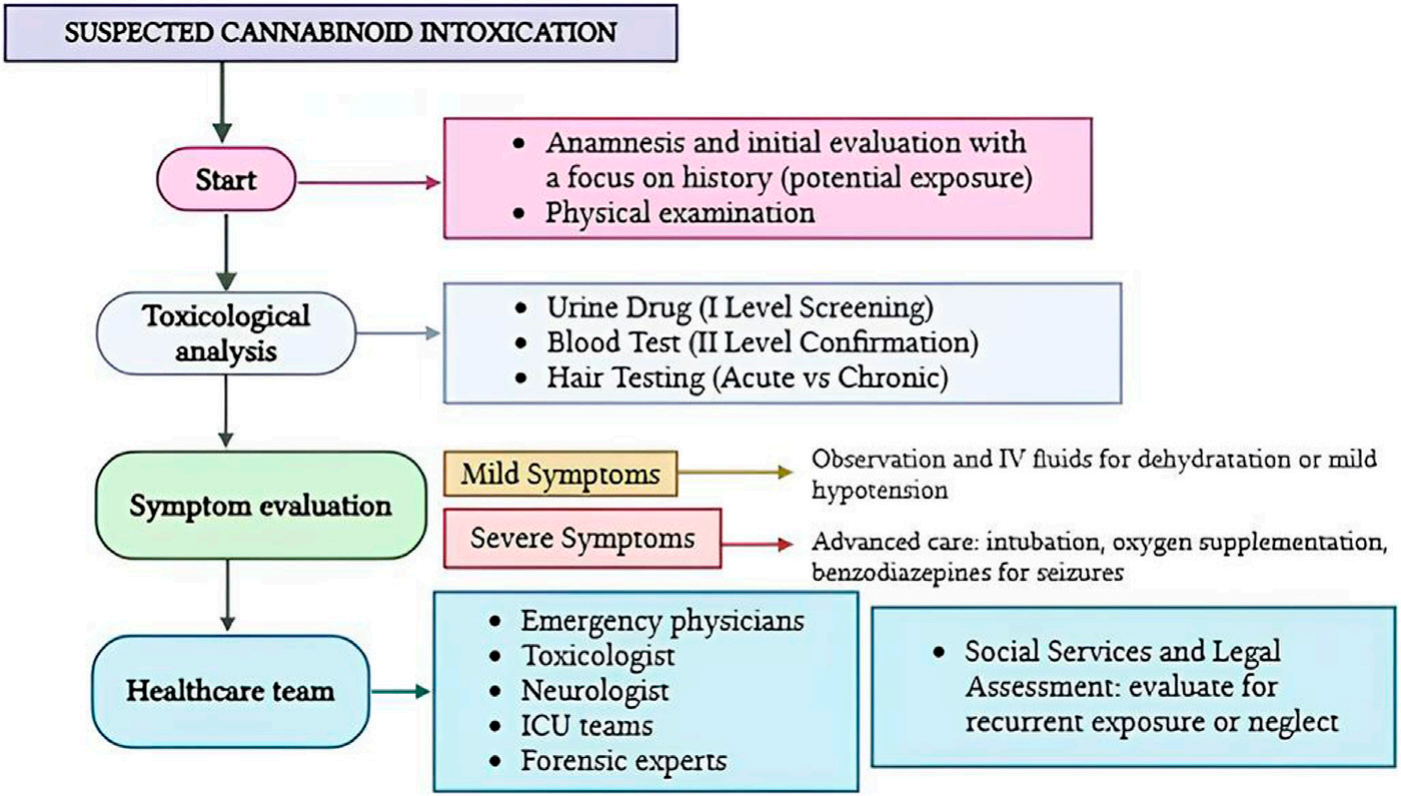
Flow diagram illustrating the patient management in cases of paediatric cannabinoid intoxication.

Currently, there are significant gaps in the literature related to evidence-based clinical management strategies, environmental and social risk factors of acute cannabis intoxication to target prevention, and support for healthcare providers (HCPs) and families. Because of increasing rates of children unintentionally ingesting marijuana products, healthcare providers who care for paediatric patients in emergency settings need to be familiar with the clinical presentation, evaluation, and management while working as a team (Figure 3).

Recognition of marijuana exposure and improved surveillance methods with standardized training for healthcare providers can lead to improved patient outcomes while avoiding unnecessary tests, monitoring and costs (Gaudet et al., 2024; Bashqoy et al., 2021; Burns et al., 2018).

## 4 Analytical toxicology of cannabinoids

Cannabinoids constitute a diverse class of chemical compounds predominantly derived from the Cannabis sativa plant. These compounds have garnered considerable scientific and clinical interest due to their complex pharmacological profiles and their increasing prevalence in both medicinal and recreational contexts (Duczmal et al., 2024). Their therapeutic applications range from pain management and anti-inflammatory treatments to seizure control, while recreational use has expanded alongside changing legal and societal attitudes toward cannabis consumption (Bridgeman and Abazia, 2017).

Given the growing accessibility and use of cannabis products, analytical toxicology has emerged as a vital discipline for accurately detecting and quantifying cannabinoids in biological matrices (Karschner, Swortwood-Gates, and Huestis, 2020). This field supports a broad spectrum of applications, including monitoring drug misuse, verifying adherence to prescribed therapeutic regimens, and ensuring safety standards in occupational and domestic environments. Advanced analytical methods not only facilitate the identification of cannabinoids and their metabolites but also enable differentiation between recent and past exposure, providing critical insights for both clinical and forensic investigations (Deidda et al., 2022).

### 4.1 The role of matrices in cannabinoid analysis

The selection of an appropriate biological matrix is a critical factor in the detection and analysis of cannabinoids, as it directly impacts both the timeframe within which cannabinoids can be identified and the interpretive value of the results obtained (Antunes et al., 2023). Each biological matrix offers distinct advantages and limitations, necessitating careful consideration based on the specific objectives of the analysis, whether clinical, forensic, or research-oriented (Kunisch et al., 2023).

Blood is widely regarded as the gold standard for assessing acute impairment associated with cannabis use (Gilman et al., 2022). Its ability to measure active THC concentrations makes it particularly useful for detecting recent consumption and correlating cannabinoid levels with physiological and behavioral effects (Burr et al., 2021). This capability is especially valuable in forensic toxicology and roadside testing for impaired driving (Fitzgerald et al., 2023). However, the utility of blood as a matrix is constrained by the rapid pharmacokinetics of THC. Following ingestion or inhalation, THC is rapidly distributed and metabolized, resulting in a narrow detection window, typically lasting only a few hours post-consumption (Hansen et al., 2024).

The transient presence of THC in blood poses challenges for retrospective analysis and may not adequately capture patterns of chronic or habitual use. Consequently, alternative matrices, such as urine, saliva, hair, and sweat, are often employed to complement blood testing, extending detection windows and providing a more comprehensive assessment of cannabinoid exposure (Zughaibi et al., 2023). Despite its limitations, blood remains indispensable for evaluating recent intoxication and impairment, particularly in scenarios requiring precise correlation between cannabinoid levels and clinical presentation. Urine testing is helpful to assess a previous cannabis use, especially in workplace screening and for drug use monitoring. However, its use is limited due to inability to determine the cannabis use in the impairment window. Oral fluid has similar characteristics: is a convenient, non-invasive method for screening but it is not a reliable method to assess recent use within the impairment window (Gregorio et al., 2024). Exhaled breath is a highly promising alternative testing medium to blood and oral fluid for detecting recent cannabis use within the impairment period. THC is typically present in breath for approximately 2 h after smoking, even among frequent users, which is shorter than the duration of impairment following cannabis inhalation (Gregorio et al., 2024). A novel testing method that integrates exhaled breath and blood analysis has been developed, enabling the identification of recent cannabis inhalation within the impairment window while minimizing false positives associated with other techniques. Ongoing research aims to refine analytical techniques and improve the sensitivity of detection methods, enhancing the reliability of cannabinoid measurements across diverse matrices (Gregorio et al., 2024).

In the paediatric population, given the immaturity of metabolic enzyme systems and differences in body composition, such as higher fat content and an underdeveloped blood-brain barrier (Weber et al., 2012), children may experience prolonged intoxication and increased sensitivity to cannabinoids. Blood tests are critical in these cases to confirm recent exposure, correlate clinical symptoms with THC concentrations, and guide emergency management strategies. However, given the rapid elimination of THC from the blood, urinalysis is often used to detect metabolites, providing evidence of exposure even after the acute phase has resolved (Qian et al., 2024).

### 4.2 Analytical techniques for cannabinoid detection

Accurate detection of cannabinoids relies on a combination of screening and confirmatory techniques. Immunoassays, including enzyme-linked immunosorbent assays (ELISA) and radioimmunoassays, are widely employed for initial screening (Tassoni et al., 2016). These assays are rapid, cost-effective, and suitable for high-throughput testing. However, their susceptibility to cross-reactivity often necessitates confirmatory analysis using chromatographic techniques (Yu et al., 2021). Moreover, although symptoms could be present, toxicological screening analyses could be negative in 4% of the cases. This phenomenon could be due to low specific cut-off ranges of the laboratory (Richards et al., 2017).

Gas chromatography-mass spectrometry (GC-MS) and liquid chromatography-tandem mass spectrometry (LC-MS/MS) are regarded as the gold standards for confirmatory testing due to their superior sensitivity, specificity, and ability to identify structural analogs. GC-MS, which requires derivatization to enhance volatility, offers excellent separation and quantification. LC-MS/MS eliminates the need for derivatization and provides enhanced detection of polar metabolites, streamlining the analytical process (Antunes et al., 2023).

Emerging techniques, such as high-resolution mass spectrometry (HRMS) and time-of-flight mass spectrometry (TOF-MS), are enhancing the detection and identification of novel synthetic cannabinoids. These approaches allow for retrospective data analysis, facilitating the discovery of new compounds and metabolites (Matey et al., 2023).

Sample preparation is a critical step in cannabinoid analysis, focusing on the removal of interfering substances and enrichment of target analytes. Traditional methods include liquid-liquid extraction (LLE) and solid-phase extraction (SPE). SPE offers advantages such as improved selectivity, reduced solvent usage, and compatibility with automated systems, aligning with the principles of green chemistry (Woźniczka et al., 2023).

Advancements in microextraction techniques, including solid-phase microextraction (SPME) and dispersive liquid-liquid microextraction (DLLME), have further improved sensitivity and minimized sample volume requirements. These techniques are particularly beneficial for handling complex matrices, such as hair and sweat (Barroso, Gallardo, and Queiroz, 2015).

### 4.3 Interpretation of results and challenges for paediatric-specific variables

The interpretation of cannabinoid concentrations is inherently dependent on the type of biological matrix analyzed, the pharmacokinetics and pharmacodynamics of tetrahydrocannabinol (THC) and its metabolites, and the timing of sample collection relative to exposure (Baglot et al., 2021). Each biological matrix provides distinct insights into cannabinoid use patterns, making their selection critical for both clinical and forensic applications. In paediatric cases, these considerations are particularly complex due to developmental differences in metabolism, distribution, and excretion, which influence the accuracy and relevance of results. Blood analysis remains the gold standard for detecting recent cannabis use, as it measures active THC concentrations, which are closely correlated with intoxication and impairment. In paediatric patients, blood sampling is particularly useful in acute intoxication cases where rapid confirmation of exposure is required to guide treatment decisions (Fernandez et al., 2011; Berdai et al., 2024). However, the short detection window of THC in blood—typically a few hours—limits its utility for retrospective evaluations, especially in delayed presentations. Moreover, paediatric patients may exhibit altered THC clearance rates due to immature hepatic enzyme systems and higher fat-to-body-mass ratios, which can lead to prolonged effects despite low blood THC levels. A recent study conducted in France on 26 paediatric cannabis intoxication cases, showed mean plasma concentrations of THC, 11-OH-THC and THC-COOH of 29 ng/mL, 21 ng/mL and 255 ng/mL respectively (Arbouche et al., 2023). Moreover, there is no strong evidence about the correlation between the degree of intoxication and THC metabolites plasma concentration. In this regard a recent study showed the presence of coma in children with blood THC concentration higher than 60 ng/mL (Mehamha et al., 2021). Although the time lapse interval is crucial to interpret THC concentration and it is frequently missing, a concentration over 50–60 ng/mL could be suggestive of the severity of poisoning (Mehamha et al., 2021). The literature also suggests that due to the highly lipophilic nature of THC, the redistribution into lipid-rich compartments, including the brain is facilitated (Ohlsson et al., 1980; Arbouche et al., 2023). Following oral administration, the slow absorption of THC could result in previously sequestered THC in the brain being gradually released. Concurrently, increased extraction of 11-OH-THC in the liver may lead to its reentry into systemic circulation (Ohlsson et al., 1980; Arbouche et al., 2023). Unlike plasma concentrations, THC levels in the brain demonstrate a strong correlation with the degree of intoxication (Ohlsson et al., 1980; Arbouche et al., 2023).

Urine analysis, on the other hand, detects THC metabolites, particularly 11-nor-9-carboxy-THC (THC-COOH), which remain detectable for days to weeks after use, depending on the frequency of exposure. This makes urinalysis well-suited for identifying prior cannabis use in paediatric cases where chronic exposure or neglect is suspected (Gonçalves et al., 2019). The usual limit of detection described for THC-COOH testing is 50 mg/mL. It is not recommended to perform urinalysis after very recent ingestion, as positive results will not occur until 4–6 h after ingestion (Alvarez et al., 2018).

However, the presence of metabolites in urine does not distinguish between acute intoxication and past use, posing challenges when correlating findings with clinical symptoms (Schuster et al., 2020). In paediatric evaluations, this limitation underscores the need to integrate toxicological results with a thorough clinical history and physical examination. Hair analysis offers the longest detection window, extending weeks to months, and is particularly advantageous in cases where chronic exposure or long-term neglect is suspected (Wang and Drummer, 2015; Claudet et al., 2020). In paediatric populations, hair analysis may be useful for forensic investigations and child protection cases, providing cumulative evidence of repeated exposure. However, its interpretation must account for factors such as age-related differences in hair growth rates, pigmentation, and the potential for external contamination, especially in cases involving passive smoke exposure. In this regard, hair from children under 3 years old is not reliable to distinguish acute from chronic administration of cannabis (Wang and Drummer, 2015; Claudet et al., 2020; Alvarez et al., 2018). Distinguishing environmental contamination, unintentional and intentional ingestion from toxicological analyses results is a great challenge. The literature suggests that THCA-A is a non-psychoactive precursor of THC, therefore a marker of environmental contamination, meanwhile THC-COOH can be considered a marker of absorption (Claudet et al., 2020). Saliva testing, due to its non-invasive nature and ability to detect recent use, is increasingly utilized in emergency settings for rapid screening. In paediatric patients, it provides a practical and less distressing alternative to blood sampling. Nonetheless, its limitations include short detection windows and susceptibility to contamination, which may compromise accuracy in cases of passive exposure (Alvarez et al., 2018).

Interpreting cannabinoid concentrations in paediatric cases involves additional complexities arising from physiological and developmental factors. Immature enzyme systems, including cytochrome P450 isoforms (CYP2C9, CYP2C19, and CYP3A4) and glucuronidation pathways, may result in slower THC metabolism and prolonged half-lives, leading to extended toxicity despite declining cannabinoid levels in blood or saliva (Doohan et al., 2021). Similarly, higher fat content in infants and young children facilitates THC sequestration in adipose tissues, contributing to delayed redistribution and prolonged effects even after initial exposure ceases (Gunasekaran et al., 2009).

Passive exposure represents another challenge, particularly in cases involving secondhand smoke inhalation. Paediatric patients living in environments where cannabis is used may test positive despite having no direct contact with the substance (Sangmo et al., 2021). Differentiating between passive and active exposure requires comprehensive history-taking and, in some cases, additional confirmatory testing. Synthetic cannabinoids further complicate interpretation due to their distinct pharmacokinetics, which often involve more potent receptor binding and unpredictable toxicological profiles (Cannizzaro et al., 2016; Roque-Bravo et al., 2023). These substances may not be detectable using standard immunoassays, necessitating more advanced chromatographic techniques, such as gas chromatography-mass spectrometry (GC-MS) or liquid chromatography-tandem mass spectrometry (LC-MS/MS). In paediatric evaluations, this variability increases the risk of misdiagnosis or underestimation of exposure severity (Namera et al., 2015).

The evolving legal status of cannabis continues to present significant challenges for analytical toxicology, particularly in the context of paediatric evaluation (Wilson and Rhee, 2022). Jurisdictions vary widely in their definitions of impairment, and permissible use, necessitating flexible and adaptable analytical protocols to address the diverse legal and clinical scenarios encountered. In paediatric cases, these challenges are compounded by the need to differentiate between accidental and intentional exposure, establish timelines of ingestion, and assess the severity of intoxication. Analytical toxicology plays a central role in addressing these complexities by providing objective evidence to guide clinical care, inform legal decisions, and support child protective interventions (Wojciechowki et al., 2024).

Workplace drug testing programs, which emphasize fairness, reliability, and compliance with regulatory standards, have long relied on validated methodologies (Plescia et al., 2021). However, the paediatric population requires additional considerations, particularly due to differences in pharmacokinetics and metabolic profiles (Hazle et al., 2022). In this context, standard analytical approaches must be adapted to account for age-specific variations, including immature enzymatic systems, altered cannabinoid metabolism, and prolonged elimination half-lives. These adaptations are critical not only for accurate interpretation but also for ensuring that results are appropriately contextualized within the developmental stage of the child (Forner-Piquer et al., 2024).

International guidelines, such as those established by the Substance Abuse and Mental Health Services Administration (SAMHSA) and the European Workplace Drug Testing Society (EWDTS), provide valuable frameworks for setting limit of detectionvalues, quality control measures, and standardized procedures for specimen collection and analysis (Cooper et al., 2011). While these protocols are primarily designed for adult populations, paediatric applications necessitate additional refinements. For instance, lower limit of detectionvalues may be required to detect low-dose exposures, and testing methods should address the influence of paediatric physiology on drug absorption, distribution, metabolism, and excretion. Furthermore, age-specific reference ranges should be incorporated to facilitate more accurate interpretation of results.

## 5 Medico-legal issues of acute cannabis intoxication in paediatric population

Acute cannabis intoxication in children represents a growing public health concern with significant medico-legal implications (Farst and Bolden, 2012; Dinis-Oliveira and Magalhães, 2013). The increased availability and normalization of cannabis due to its legalization in many countries have led to an alarming rise in paediatric exposure cases (Leubitz et al., 2021). Such incidents are not only medical emergencies, but also potential indicators of parental neglect, insufficient supervision or abuse (Walsh et al., 2003).

### 5.1 Definitions

Child maltreatment/abuse is defined as: “All forms of physical and/or emotional abuse, sexual abuse, neglect or negligent treatment, as well as sexual or other types of exploitation, which result in actual or potential harm to the child’s health, survival, development, or dignity, within the context of a relationship of responsibility, trust, or power” (World Health Organization, 1999; Krug et al., 2002; World Health Organization, 2006).

Child maltreatment/abuse is an extremely complex phenomenon, sometimes reported by the child themselves, a family member, or strangers, but more often suspected during a medical evaluation conducted for another reason, based on the child’s history, behaviors, and objective findings (Massullo et al., 2023).

World Health Organization distinguishes four kinds of child maltreatment: physical abuse, sexual abuse, emotional and psychological abuse, neglect (World Health Organization, 2006). Substance use can have a causal link to child abuse: substances may impair the alertness and emotional state of the alleged perpetrator, affecting vigilance and potentially leading to violent actions. In the absence of proper supervision, a child may unintentionally ingest drugs. Additionally, a caregiver may forcibly administer substances, sometimes to facilitate sexual abuse (Table 2).

**TABLE 2:** Summary of definitions about child abuse and neglect (World Health Organization, 2006).

Term	Definition
Child maltreatment	All forms of physical and/or emotional maltreatment, sexual abuse, neglect or negligent treatment, as well as sexual or other exploitation, that cause actual or potential harm to a child’s health, survival, development, or dignity within the context of a relationship of responsibility, trust, or power
Physical abuse	Intentional use of physical force against a minor that causes or has a high probability of causing harm to their health, survival, development, or dignity
Sexual abuse	Involvement of a minor in sexual acts that they do not fully understand, cannot give informed consent to, are not developmentally prepared for, or that violate laws or social taboos
Emotional and psychological abuse	Includes both isolated incidents and ongoing situations where a caregiver fails to provide an appropriate and supportive environment for the child’s development. Acts in this category have a high likelihood of causing harm to the minor’s physical and mental health, as well as their physical, mental, spiritual, moral, and social development
Neglect	It includes both isolated situations and a repeated, ongoing neglectful attitude by parents or other family members who, despite being capable, fail to ensure the development and wellbeing of the child
Neglectful unintentional intoxication	A child is inadvertently exposed to or ingests cannabis or cannabis-containing products as a result of inadequate supervision or failure of caregivers to ensure a safe environment
Nonneglectful unintentional intoxication	Unintentional exposure not due to neglect
Intentional intoxication	Intent to cause child intoxication
Drug-facilitated sexual assault	A form of sexual assault in which a perpetrator uses alcohol, drugs, or other substances to incapacitate a victim, reducing their ability to resist or give informed consent to sexual activity

Paediatric cannabis intoxication, being one of the most frequent and increasingly prevalent conditions due to the widespread use and legalization in certain geographic areas, should be considered in the differential diagnosis of suspected child abuse (Tendler et al., 2020). Management and applied methodologies must aim to confirm or rule out the aforementioned conditions to make appropriate clinical and legal decisions for the health and safety of the patient. Familiarity with the terminology and diagnostic approach constitutes an essential cultural and professional foundation for every healthcare professional.

### 5.2 Legislation

The need to identify maltreatment/abuse arises not only from specific legal obligations but also from the necessity to interrupt the cycle of violence and re-victimization (Sege et al., 2011). Any abusive event can cause short- and long-term harm to a child’s health and psychological wellbeing, affecting their emotions, behavior, and ability to develop interpersonal relationships. The harm becomes more severe the longer the abuse remains hidden and unrecognized, leading to repeated episodes and delayed or avoided protective intervention (Hussey et al., 2006).

Child neglect laws in the United States are state-specific but uniformly emphasize protecting minors from harm caused by inadequate supervision. When a child experiences cannabis intoxication due to a parent’s failure to secure cannabis products, this may be classified as child endangerment (McDaniel, 2020). For instance, in California, Penal Code Section 273a penalizes conduct that places a child at risk of harm, which can include allowing access to cannabis edibles or other products. Similarly, in states like Colorado and Washington, where cannabis is legal, specific regulations mandate childproof packaging to mitigate such risks. Noncompliance can lead to civil or criminal liability for parents.

In Italy, the Penal Code addresses child neglect under Article 591, which prohibits abandoning a minor or any individual unable to care for themselves. Article 572 prohibits mistreatment within the family and the abuse of disciplinary and corrective measures. Parents can face criminal charges if their failure to supervise leads to cannabis intoxication in a child. Italian courts also consider environmental factors, such as household dynamics and parental behavior, when assessing neglect. Additionally, Italy’s child welfare system actively involves social services in cases where neglect is suspected, providing an additional layer of protection. Across Europe, legal responses to child neglect and ill-treatment share similarities but vary in implementation. In France, Article 227-17 of the Penal Code penalizes parental behavior that compromises a child’s health or safety. Germany’s Civil Code and Penal Code obligate parents to provide adequate care and supervision, with neglect leading to potential criminal sanctions. These frameworks underscore a collective commitment to prioritizing child welfare, particularly in cases involving substance-related harm.

Whether cannabis intoxication in children, even accidental intoxication, should be considered as indicating insufficient supervision by parents is unclear. The definition of inadequate parental supervision varies between clinicians and both police and child protection social workers are concerned about the presence of children in homes where parents are addicted to cannabis (Pélissier et al., 2014).

It is important to emphasize that the obligation to report child abuse and neglect varies across jurisdictions. Differences exist regarding the type of maltreatment that must be reported and, in some cases, the source of the maltreatment. These differences stem from the numerous definitions of child abuse and neglect, as well as the various legal requirements. Most countries automatically involve the judicial system in cases of sexual abuse, serious injuries, or death, even when only suspected, as is the case in Italy (Tjaden and Thoennes, 1992). In France, a severe injury is required for healthcare professionals to report the case to judicial authorities (Pélissier et al., 2014). In a recent review, Pietrantonio et al. (2013) noted that some legislations mandate healthcare professionals to report child abuse and neglect, such as in the United States, Canada, Australia, Argentina, Israel, Poland, and Sri Lanka. In contrast, other countries, including the United Kingdom and New Zealand, do not require healthcare professionals to report concerns about child abuse and neglect.

Child abuse and neglect are underrecognized and underreported in emergency departments (EDs), but the reasons behind this phenomenon have not been thoroughly investigated. Despite being in a privileged position to report abuse, healthcare professionals account for only a small proportion of child abuse reports to social services. A study conducted in Canada revealed that the majority of reports (24%) came from school personnel, while only 5% were made by hospital staff (Trocmé, 2010). A similar study in the United States showed that reports were most commonly made by law enforcement personnel (16.7%), followed by school staff (16.4%), social workers (11.5%), and finally healthcare professionals (8.2%) (U.S. Department of Health and Human Services, 2012). The Child Abuse Recognition and Evaluation Study (CARES), a large national prospective study conducted in the United States, examined decision-making by primary healthcare providers (PHCPs). The study revealed that 27% of PHCPs did not report injuries to Child Protective Services (CPS), even though they believed the injuries were “likely” or “very likely” caused by child abuse (Flaherty et al., 2008; Alvarez et al., 2010). A *post hoc* evaluation of some CARES visits was conducted by child abuse experts and PHCPs. This evaluation found that experts agreed with the reporting decisions made by PHCPs in 84% of cases. However, experts also determined that 21% of the non-reported cases should have been reported. The literature suggested two main groups of reasoning for underreporting a child abuse: lack of knowledge and decision not to report influenced by other factors (Flaherty et al., 2008; Sege and Flaherty, 2008).

There is a limited awareness among healthcare professionals regarding the early identification of maltreatment (Pietrantonio et al., 2013; Flaherty et al., 2008; Sege and Flaherty, 2008). Studies highlight both a desire to implement specific training programs and the effectiveness of educational interventions in increasing the reporting of abuse and/or maltreatment (Ward et al., 2004; Alvarez et al., 2010).

A study conducted in three paediatric emergency departments in Connecticut, United States, identified the most common factors contributing to the failure to report abuse to judicial authorities. These factors included a lack of expertise, a desire to believe the caregiver, personal biases, and concerns about making an incorrect reporting decision (Tiyyagura et al., 2015). A national survey conducted in the United States highlighted additional factors contributing to underreporting, including legal concerns and the potential loss of the relationship between the child and their family (Flaherty et al., 2006). Moreover, in case of substance intoxication, there are no validated methods to assess a lack of supervision and a neglectful cannabis intoxication and consequently to report adequately to the children protective services and the Judicial Authority.

Healthcare professionals must be aware of the relevant legislation, appropriately identify the problem, and report it to the competent authorities. Raising awareness and providing training in this area are essential, given the tendency to underestimate and inadequately report the issue.

### 5.3 Epidemiology and literature data

Acute cannabis intoxication in the paediatric population has become a significant public health concern, especially in contexts where the substance’s legalization and increasing accessibility intersect with child maltreatment and neglect.

The incidence of acute cannabis intoxication in children has risen significantly in regions where cannabis has been legalized. Recent studies report a notable increase in paediatric emergency department (ED) visits for cannabis-related issues following legalization in various states in the United States (Freisthler, Gruenewald, and Wolf, 2015; Graham et al., 2020). A study conducted over a 10-year period (2006–2016) found an increase in marijuana intoxication reports to the National Poison Data System from 9.3 to 68.1 calls per 100,000. For instance, Wang et al. found that Colorado experienced a spike in paediatric cannabis exposures after legalization, with an increase in unintentional ingestions among children under 6 years of age (Wang et al., 2016).

Geographically, the prevalence of paediatric cannabis intoxication mirrors patterns of legalization and societal acceptance. North America, particularly the United States and Canada, shows the highest incidence due to early adoption of cannabis legalization. European nations have lower reported rates, likely reflecting stricter regulations and slower adoption of legalization policies (Freisthler, Gruenewald, and Wolf, 2015; Myran et al., 2024).

Neglectful intoxication often arises from unsafe storage of cannabis products, parental substance use, and broader socioeconomic factors (Freisthler, Gruenewald, and Wolf, 2015; U.S. Department of Health and Human Services, 2009). A study by Onders et al. emphasized that edible cannabis products, such as gummies and chocolates, pose a particular risk due to their appeal to children and inadequate childproof packaging (Onders et al., 2016). Parental negligence in safely storing these products has been identified as a critical factor. Despite a recent study conducted in Colorado on parents using marijuana for medical purposes highlighting an improvement in their perception of parenting skills (Thurstone et al., 2013), it is widely demonstrated that cannabis use impairs attention, short-term memory, and motor coordination. These effects can undermine a parent’s supervisory abilities and are consistent with neglect (Freisthler, Gruenewald, and Wolf, 2015; U.S. Department of Health and Human Services, 2009).

Economic and social stressors also play a role. Neglectful supervision often coincides with caregivers’ own substance abuse issues or mental health challenges. The COVID-19 pandemic exacerbated these vulnerabilities, with increased parental cannabis use reported during lockdowns (Zhang et al., 2022). Recent literature documented heighteness of paediatric intoxication during the pandemic, underscoring the interplay between increased cannabis availability, caregiver stress, and neglectful behaviors (Zhang et al., 2022).

Conversely, some evidence suggests that legalization can improve awareness and safety practices through public health campaigns (Hinckley et al., 2021). However, these benefits are often undermined by inconsistent regulations regarding packaging and labeling the COVID-19 pandemic created an environment conducive to increased paediatric cannabis intoxication (Freisthler, Gruenewald, and Wolf, 2015; Zhang et al., 2022). Isolation, economic hardship, and the closure of schools contributed to higher stress levels among caregivers, potentially leading to greater cannabis use and decreased vigilance. It was demonstrated a sharp rise in ED visits for paediatric cannabis intoxication during the pandemic, correlating with increased sales of cannabis products (Freisthler, Gruenewald, and Wolf, 2015; Zhang et al., 2022).

The relationship between cannabis intoxication and child maltreatment is complex and often influenced by legal definitions. Laws in many jurisdictions categorize neglectful exposure to cannabis as a form of child maltreatment, mandating reporting by healthcare professionals. Underreporting remains a challenge, often due to healthcare providers’ uncertainty about legal thresholds for reporting. Evidence from the literature suggests that children presenting with cannabis intoxication may be at higher risk for other forms of maltreatment, such as physical abuse or neglect (Freisthler, Gruenewald, and Wolf, 2015). The presence of cannabis intoxication should prompt a thorough assessment of signs of maltreatment and neglect in the patient, child’s home environment and parental behaviors.

## 6 Public health interventions

Effective strategies to reduce the risk of acute cannabis intoxication in children and adolescents require a comprehensive, multi-pronged approach. This includes educational initiatives, regulatory policies, market controls, and improved awareness among healthcare and social service professionals.

Educational programs in schools play a crucial role in preventing adolescent cannabis use by providing students with accurate information about the associated risks. Research by Donnelly et al. (2022) highlights the effectiveness of school-based prevention programs that incorporate evidence-based strategies, such as resistance skills training and normative education. These programs help dispel misconceptions about cannabis and reduce the likelihood of initiation among youth.

Strict regulatory measures and market controls are also essential for limiting minors’ access to cannabis. Conerney et al. (2024) emphasize the importance of stringent packaging, labeling, and potency restrictions on cannabis edibles to prevent accidental ingestion by children. For example, the Cannabis Act in Canada mandates child-resistant packaging and specific health warnings (Government of Canada, 2019). Similar regulations exist in U.S. states such as Alaska, Colorado, and Washington. In the state of Massachussets, there was an increased frequency of pediatric edible cannabis exposure after cannabis legalization (Kaczor et al., 2021). Research suggests that well-executed mass media and school-based awareness campaigns have significantly reduced liquid nicotine intoxications in children, indicating that similar strategies could be effective for cannabis (Chang et al., 2019; Conerney et al., 2024). However, studies assessing the impact of public health initiatives on pediatric cannabis intoxication remain limited (Gaudet et al., 2024).

Further regulatory efforts, including restrictions on youth-targeted advertising and stringent age verification processes, also help curb underage access. Despite these measures, underreporting remains a significant issue, as shown by data from the Canadian Paediatric Surveillance Program (2019). A recent systematic review explored the relationship between medical cannabis use, public health, and safety (Sznitman and Zolotov, 2015). The findings indicate no clear link between the legalization of cannabis for therapeutic purposes (CTP) and increased cannabis use in the general population. Anderson et al. (2013) found a correlation between CTP legalization and reduced alcohol consumption, although the underlying mechanisms remain unclear. Another study reported a decrease in suicide rates among males in states that legalized CTP, potentially due to improved coping mechanisms for stress (Anderson et al., 2014).

Healthcare professionals and social service providers must be adequately trained to recognize and manage cannabis-related concerns. Bernstein et al. (2009) emphasize the importance of screening and brief intervention programs in pediatric emergency settings to identify at-risk youth and provide timely support. Training healthcare providers to recognize signs of cannabis intoxication and dependence ensures prompt and appropriate responses.

Standardized protocols for managing cannabis-related incidents in healthcare and emergency settings are critical. These should include guidelines for recognizing symptoms, providing appropriate medical care, and referring patients to educational or rehabilitative resources as needed. Donnelly et al. (2022) advocate for the implementation of such standardized procedures to improve response efficiency. A holistic public health strategy—integrating education, strict regulations, market oversight, professional training, and standardized intervention protocols—is essential for reducing adolescent cannabis use and preventing pediatric intoxication. The available literature supports the effectiveness of these measures in minimizing the adverse effects of cannabis on young people and fostering a safer public health environment.

## 7 Conclusions and future perspectives

This narrative review synthesizes current evidence on the toxicological, clinical, and medico-legal dimensions of acute cannabis intoxication in the paediatric population. The pharmacokinetics of cannabinoid ingestion in children are significantly influenced by the immaturity of their gastrointestinal tract and metabolic enzyme systems, resulting in altered oral bioavailability. With the increasing availability of high-THC cannabis products and the relatively lower body mass of young children, even small amounts of ingestion can lead to disproportionately high plasma THC levels. Clinical data indicate that THC-related effects in paediatric patients typically emerge within 2 hours of ingestion, with more severe symptoms developing within 4 hours. Consequently, asymptomatic children should be closely monitored for delayed onset of clinical manifestations.

Age-related pharmacokinetic factors, including developmental variability in CYP and UGT enzyme activity, contribute to an increased CNS distribution of THC and a prolonged half-life in children. Cannabinoid effects are mediated primarily through CB1 receptor activation, which plays a central role in regulating key physiological processes. The ECS undergoes significant developmental changes, with marked differences in cannabinoid receptor expression and distribution across fetal, neonatal, and adult brains. During neurodevelopment, CB1 receptors exhibit unique expression patterns, including transient localization in brainstem regions critical for neurovegetative functions. These developmental dynamics likely explain children’s heightened sensitivity to THC’s neurological and neurovegetative effects, often resulting in more severe outcomes compared to adults.

Further experimental research is needed to elucidate the pharmacodynamic aspects of paediatric cannabis intoxication and provide clinicians with robust evidence-based guidance. Acute cannabis intoxication in children primarily presents with neurological symptoms (e.g., lethargy, ataxia, hypotonia, and coma) and cardiovascular effects (tachycardia, hypotension). Symptom severity and duration depend on individual patient factors as well as the type and method of cannabis exposure. Treatment is largely symptomatic and supportive, focusing on vital sign monitoring and acute symptom management. Misdiagnosis or underdiagnosis is common, often leading to suboptimal care and missed opportunities to identify unsafe family or social environments.

Healthcare professionals managing such cases require heightened awareness and specialized training. Poison Center consultation is strongly recommended in severe intoxications to ensure optimal management. Diagnosing cannabis intoxication in children can be particularly challenging due to unreliable or incomplete medical histories, compounded by caregivers’ reluctance to disclose potential exposures out of fear of legal or social repercussions. In such instances, toxicological analysis becomes indispensable. It helps clinicians confirm exposure, identify unexpected toxic agents, and rule out alternative diagnoses (Ghandi et al., 2024).

The reliable detection of cannabinoids involves integrating screening methods with confirmatory analytical techniques. Urine immunoassay testing is widely considered an helpful tool to assess previous exposure, becoming positive within 3–4 h of ingestion. However, this method is prone to false positives. Plasma THC concentration, when measured close to the event, offers valuable insights into the quantity ingested and the correlation between exposure and clinical outcomes in the impairment window. Hair analysis, while useful for distinguishing between acute and chronic use, is susceptible to various biases. Interpretation of toxicological results should be undertaken by expert analytical toxicologists in collaboration with multidisciplinary teams to ensure accurate diagnosis and identify potential cases of neglect or abuse. This may involve adjusting testing thresholds for paediatric physiology and incorporating age-specific reference ranges.

The rising incidence of acute cannabis intoxication in children underscores the urgent need for targeted public health interventions and stricter regulatory frameworks. Preventive measures such as child-resistant packaging, public education campaigns, and cannabis use screening during pregnancy are essential to mitigate risks. Clinicians should consider THC exposure in the differential diagnosis of children presenting with unexplained neurological, immune, or metabolic symptoms.

Addressing this issue demands a comprehensive strategy, including enhanced caregiver education about safe storage and the risks of cannabis exposure, improved packaging regulations for cannabis products, and specialized training for healthcare professionals to recognize and manage intoxication effectively. Collaborative policy approaches should balance legalization with robust child protection measures, fostering connections between healthcare providers, social services, and judicial authorities.

Future research should prioritize longitudinal studies to deepen our understanding of the pharmacological and analytical complexities of paediatric cannabis intoxication, its long-term consequences, and its intersection with child maltreatment. Bridging these knowledge gaps will require coordinated efforts across medical, legal, and social service systems to safeguard vulnerable paediatric populations from cannabis-related harm. Protecting children from these risks is both a medical imperative and a societal responsibility.
